# Reputation and Competition in a Hidden Action Model

**DOI:** 10.1371/journal.pone.0110233

**Published:** 2014-10-17

**Authors:** Alessandro Fedele, Piero Tedeschi

**Affiliations:** 1 School of Economics and Management, Free University of Bozen/Bolzano, Bozen/Bolzano, Italy; 2 Department of Economics and Finance, Università Cattolica del Sacro Cuore, Milan, Italy; Center of nonlinear, China

## Abstract

The economics models of reputation and quality in markets can be classified in three categories. (i) Pure hidden action, where only one type of seller is present who can provide goods of different quality. (ii) Pure hidden information, where sellers of different types have no control over product quality. (iii) Mixed frameworks, which include both hidden action and hidden information. In this paper we develop a pure hidden action model of reputation and Bertrand competition, where consumers and firms interact repeatedly in a market with free entry. The price of the good produced by the firms is contractible, whilst the quality is noncontractible, hence it is promised by the firms when a contract is signed. Consumers infer future quality from all available information, *i.e.*, both from what they know about past quality and from current prices. According to early contributions, competition should make reputation unable to induce the production of high-quality goods. We provide a simple solution to this problem by showing that high quality levels are sustained as an outcome of a stationary symmetric equilibrium.

## Introduction

A high-quality product's reputation is a crucial aspect when quality is hard to measure. In this paper we develop a pure hidden action model of reputation, where only one type of seller is present in the market who can provide goods of different quality [1]. As mentioned in the abstract, consumers and symmetric competitive firms interact repeatedly and entry is free. The price of the good produced by firms is contractible. By contrast, quality is noncontractible, hence it is just promised by firms when contracts are signed.

Firms' incentive not to cheat, *i.e.*, not to produce a-lower-than-promised quality level, is based on the following mechanism. Cheating entails the expected cost of losing market share in the future due to the existence of a signal about quality. The signal is imperfect and public in that either all consumers receive it with some probability, or nobody detects cheating. Put differently, clients are generally not able to discover a firm's opportunistic behavior because of imperfect observability of quality. Yet if someone observes low quality, this piece of information becomes public through, *e.g.*, word of mouth communication, specialized publications, forums and discussion groups on internet. The Ebay system of feedbacks, *i.e.*, the ex-post evaluation of sellers (and buyers) made by the counterpart, is a real-world example of the public signal we have in mind; Tripadvisor is another one. Consumers do not repeat the purchase after receiving the signal. Furthermore, they can anticipate whether a given combination price-quality is incentive compatible, *i.e.*, such that firms find it profitable not to cheat. This amounts to say that consumers infer future quality from all available information, *i.e.*, both from what they know about past quality (the probability of receiving the public signal) and from the observation of current contracts (the agreed-upon price and the promised quality of the good).

We find a stationary Bertrand equilibrium where firms end up with positive profits and provide high-quality goods. High quality is intended as a level strictly above a minimum possible level. In turn, the minimum can be referred to as a level below which under-provision of quality can be easily verified by a Court. Profits are positive because the firms' incentive compatibility (IC henceforth) constraint commands the so-called quality premium, without which firms would produce minimum-quality goods. Finally, we generalize the analysis by verifying that our findings are robust to three extensions of our framework.

### Related literature on reputation

The literature on reputation follows two related, but distinct, strands. One studies the social role of reputation and its relationship with cooperation, trust, and trustworthiness [2]. Some of the most recent results can be found in [3] and in the literature quoted therein. Our paper deals with the other stream of literature, reputation in markets, whose aim is to study the effect of reputation on concentration, entry, prices, and, especially, service and product quality. To the best of our knowledge, no other paper found a stationary Bertrand equilibrium with high quality and positive profits in a pure hidden action model, where entry is free, firms do not collude, and consumers evaluate noncontractible quality from all available information.

Seminal research showed that in on-going relationships clients can react to a monopolistic firm's choice of providing low quality by not repeating their purchase [4]. This reaction constitutes a punishment for the firm because providing high quality commands positive profits, as in our framework. Later contributions extended the analysis to a competitive setup and proved that the quality premium is just sufficient to cover the higher costs of quality [5], [6]. As a result, firms end up with zero profits. The mechanism in [5] is as follows. Consumers are supposed to infer future quality only from the observation of past levels and to underestimate quality of new goods. Accordingly, new firms are obliged to sell high-quality products at less than cost in order to gain market share. This initial investment in reputation is just compensated by a future flow of positive profits representing the quality premium.

Interestingly, this mechanism would disappear if consumers inferred future quality also from current prices. Suppose a new firm tries to gain market share by adopting the following non-stationary strategy. It reduces quality and the short-run price of its product so that consumers are better-off compared with the competitors' offers. At the same time, the firm sets the future price in such a way that the quality premium is preserved along with its long-run incentive to produce an above-minimum quality. In this way, consumers are convinced about the high quality of the good. The short-term undercutting strategy is profitable since the firm is able to gain market share and, at the same time, preserve the future quality premium. This reasoning leads to the famous objection raised by Joseph Stiglitz [7]. Competition with free entry should eliminate any quality premium, making reputation unable to induce the production of high-quality goods.

This side-effect of competition does not occur in our equilibrium. Indeed, any undercutting strategy (lower price given the equilibrium quality, or greater quality given the equilibrium price) leads to a market share reduction, rather than increase, because consumers anticipate a violation of the firms' IC constraint. As a result, such a strategy is not profitable.

One solution to Stiglitz's objection came from a more recent contribution, which relies upon a mixed (both hidden action and hidden information) model with good and bad firms [8]. Good firms have a technological advantage in producing high quality. Quality is also affected by firms' effort choice and some randomness in a repeated market interaction. At equilibrium all firms who under-performed in quality are kicked out of the market, good firms are induced to invest in quality to avoid being pushed out of the market and profits might be positive. Reputation is thus valuable.

The Stiglitz's problem appears to be particularly severe in pure hidden action frameworks, unless consumers' beliefs on quality are conditioned only to past levels, [9], [10]. Indeed, in the absence of collusion, firms are shown to gain by cutting prices when beliefs are conditioned not only to past quality but also to current prices [11]. This confirms Stiglitz's objection. The result of high quality with "perfectly rational" beliefs is obtained when high costs of changing suppliers are imposed, which are instead absent in our framework [12]. By introducing the possibility of collusion among firms, an oligopolistic market structure is shown to sustain high quality, since firms are punished by rivals when lowering price and by consumers when cutting quality [13]. By contrast, high quality can be sustained in markets where the degree of product substitutability is either very low or very high, when a model with both vertical and horizontal differentiation is considered [14].

## Materials and Methods

No materials have been used to conceive and write this paper. The only method consists in mathematical analysis to solve a theoretical economic model, whose basic features are as follows. We consider an economy with a continuum of consumers of measure one and 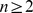 symmetric firms that provide a good. Each consumer buys at most one unit of the good, in which case she is characterized by the following utility function,
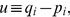
(1)where 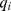 and 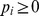 are quality level and price, respectively, of the good supplied by firm 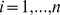. We let 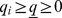, where 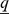 denotes the minimum possible level of quality; as mentioned, one can think of a level below which under-provision of quality can be easily verified by a Court.

Firm 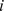 is characterized by the following profit function,
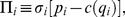
(2)where 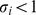 denotes the fraction of consumers served by firm 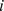 and 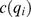 the unit cost of quality 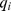, with 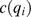 twice differentiable, 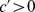, and 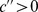.

## Results

Consumers and firms play the following one-shot competition game: (i) firms compete à la Bertrand by making simultaneous offers of 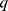 and 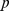; (ii) each consumer either selects the preferred contract or refuses to purchase; (iii) the accepted contracts are implemented.

### Contractible Quality

Suppose that quality 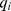 is contractible. We first solve the following problem: a representative consumer maximizes her utility 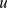 subject to firm 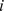
's participation constraint 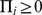. We then show that the equilibrium contract of the one-shot competition game is given by the solution to the above problem.

Before proceeding we define the sum of a consumer's utility plus firm *i*'s profit on a single contract,
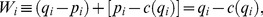
(3)as the welfare generated by each contract proposed by firm *i*. The level of quality that maximizes 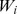 is referred to as efficient.

### Lemma 1

*The equilibrium contract* 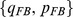 *when quality is contractible has the following features: (i) firms get zero profits; (ii) the level of quality is efficient; (iii) consumers accept the contract. In symbols:*

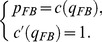
(4)


### Proof

Contract (4) is the solution to the following problem:
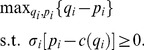
(5)


The Lagrangian is
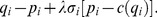
(6)


The first order conditions with respect to 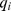 and 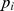 are
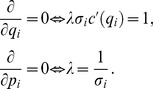
(7)


The constraint is hence binding at the optimum. Substituting 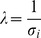 into 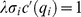 yields the result.

To prove that the 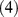 is the equilibrium contract when firms compete à la Bertrand and 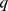 is contractible, it is sufficient to invoke a Bertrand undercutting argument. ▪

### Noncontractible Quality

We now relax the assumption of quality contractibility. This means that the contracts cannot be conditioned on 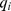. Since firm 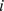
's profits, 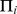, are decreasing in 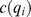, and therefore in 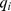, firm 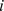 has an incentive to supply the minimum level of quality, 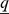, when implementing a contract 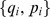.

We replicate the analysis of Lemma 1 by studying the above-described one-shot competition game under the assumption, however, that quality is noncontractible.

### Lemma 2

*The equilibrium contract* 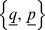 *when quality is noncontractible has the following features: (i) firms get zero profits; (ii) the level of quality is minimal; (iii) consumers accept the contract. In symbols:*

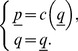
(8)


### Proof

The optimal contract is the solution to the following problem:
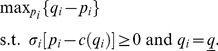
(9)


Plugging 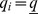 in the objective function yields 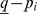, which is decreasing in 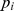. The constraint is therefore binding. Solving 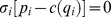 for 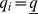 yields the result.

To prove that the 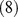 is the equilibrium contract when firms compete à la Bertrand and 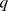 is noncontractible, it is sufficient to invoke a Bertrand undercutting argument. ▪

We let 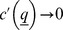, so that 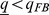 and contract 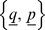 in 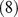 is not efficient, *i.e.*, it does not maximize the welfare generated by each single contract. We can conclude that the equilibrium contract when quality is noncontractible entails unexploited gains from trade.

### Reputation

We investigate whether reputation helps mitigate the issue of unexploited gains from trade due to quality noncontractibility. To this aim, we abandon the one-shot competition game described at the beginning of this section to consider a repeated interaction among infinitely lived consumers and firms. We assume that quality is observed by consumers when they receive a public signal, which we describe below. We first study the contracting problem between a representative firm and its customers. In order to provide an appropriate benchmark for the subsequent analysis of competition, consumers are assumed to have full bargaining power. This is the same hypothesis behind the proofs of Lemmas 1 and 2.

The fraction of consumers served by firm 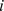 at time 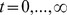 is denoted with 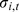. In each period 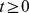, the contracting between firm 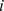 and its customers takes place according to the following timing:

- a representative consumer offers a contract 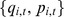 to firm 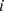;
- firm 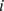 either accepts the contract or refuses it; quality 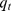 is noncontractible, hence it is promised by firms;
- firm 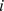 selects a quality level 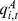 for each consumer, where superscript 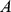 stands for actual; we denote with 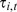 the share of consumers who enjoy a quality level lower than the promised level, 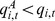, that is, cheated consumers;
- Nature selects the following public signal: with probability 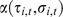 all consumers receive a signal of bad quality; with probability 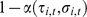 no consumer receives the signal;
- if consumers receive a signal of bad quality, they know that firm 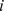 cheated somebody; they then decide whether to buy again from firm 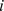 or not.

The above timing depicts a moral hazard model, where the hidden action is the actual level of quality provided by firm 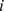 after the contract is signed.

We introduce the following restrictions on the public signal probability 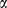:

### Assumption 1



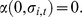



### Assumption 2


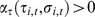
 *and* 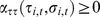, *where the subscripts of* 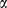 *denote partial derivatives.*

### Assumption 3



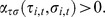



According to Assumption 1 no signal is conveyed if firm 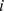 cheats no consumer, that is, we rule out the possibility that non-cheated consumers send a signal of bad quality. This hypothesis is quite reasonable. However, there may be real-world situations in which false and/or erroneous signals of bad quality are conveyed. An example of false signals is given by the case of Ebay. Evidence was found that (negative) feedbacks were used to threaten the counterpart with the aim of obtaining better contractual conditions. To take this aspect on board, in Section "Discussion" we relax Assumption 1 by introducing an alternative public signal probability 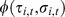, with 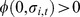.

Assumption 2 simply states that probability 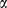 is increasing and nonconcave in the fraction 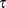 of cheated consumers.

The meaning of Assumption 3 is as follows. If firm 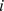 decides to cheat an additional fraction of consumers, that is, to increase 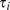 the probability that all consumers receive the signal increases since 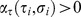. Such a variation, in turn, rises with the market share because 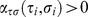. Put differently, information regarding bigger firms is supposed to propagate at a faster rate. There exists indirect evidence of the validity of our assumption in finance and management literature, where information on the accounts of big firms is thought to circulate before its disclosure [15], [16]. This may mean that big firms are subject to closer scrutiny than smaller ones on the side of the public, although we cannot exclude alternative explanations, such as the strategic use of information leaks. There is also evidence that the number of analysts following big firms is typically higher [17]. This implies that privately gathered information about big firms is likely to be more abundant. In addition, there are theoretical contributions which show that both information and word of mouth reputation are more valuable for big firms [18], [12]. We finally mention a survey on the role of risk managers in protecting corporate reputation [19]. Evidence is found that bigger companies undertake more reputational risk management activities, perhaps reflecting a greater consideration for the value of reputation.

Obviously, we cannot exclude opposite situations where information regarding bigger firms circulates at a slower rate. To take into account this scenario, in Section "Discussion" we relax Assumption 3 by introducing an alternative public signal probability 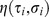, with 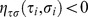.

At time 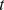 the discounted value of firm *i*'s profit is
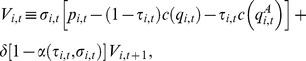
(10)where 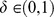 is the discount factor. When cheating 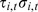 consumers at any time 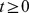, firm 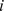 saves the amount 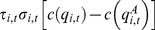, but incurs the expected loss 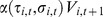 of future profits, provided that no consumer repeats the purchase when receiving the signal of bad quality. Point 5 of Proposition 1 below shows this is the consumers' equilibrium behavior.

As one can see by inspecting (10) the choice of 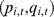 affects 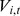 but not 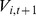. Only 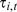 has a dynamic effect on firm *i*'s profits. However, 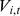 turns out to have a stationary structure, *i.e.*, 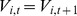 for all 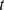, if problem 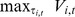 has a stationary solution, that is, 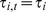 for all 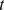. This is the case because in Lemma 3 below we compute the conditions for which firms find it profitable not to cheat any customer; in symbols, 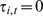 for any firm 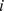 at any time 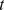.

### Lemma 3

*In a stationary strategy and for any given market share* 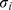 *, firm* 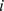 *decides not to cheat any consumers if and only if its profits on each contract are relatively high. In symbols,*

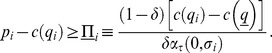
(11)


### Proof

Expression (10) decreases with 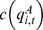, hence the optimal deviation is setting 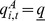, in which case 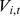 can be rewritten as
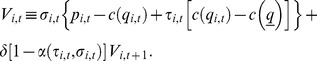
(12)


Note that
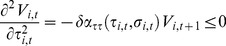
(13)according to Assumption 2. As a consequence, firm 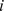 will not cheat if and only if
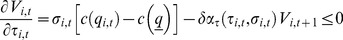
(14)at 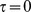. We assume that our dynamic model is stationary, 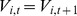, and we then check that a stationary solution is admissible. Putting 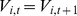 with 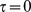 in (12), recalling that 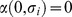 under Assumption 1, and omitting subscript 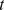 yields



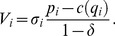
(15)Plugging the above value of 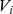 into (14) yields
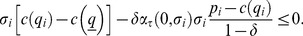
(16)


Rearranging gives 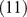. ▪

Condition 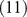 defines the firms' incentive compatibility (IC) constraint, which states that firms must make positive profits on each contract in order not to cheat any consumer. If profits were nought there would be no quality premium, hence no fear of foregoing future profits. In that case, firms would not be induced to behave. To illustrate the IC constraint 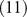 we rewrite it as
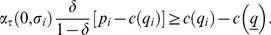
(17)


The left hand side of 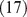 denotes the long-run expected loss of cheating an additional consumer when 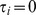: the increase in the probability that firm 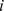 is detected is 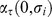, in which case it loses the per-contract profits in all future periods, 
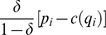
. The right hand side of 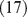 denotes the short-run gain of cheating, due to the fact that firm 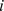 produces the minimum quality 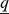 instead of 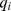. The expected loss of cheating is larger than the gain when 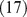 is fulfilled, in which case firm 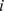 finds it profitable not to cheat any clients.

We are now able to compute the optimal contract with reputation as a solution to the following problem. Since the model is stationary, a representative consumer selects 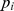 and 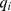 to maximize her single-period utility 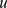 subject to firm 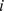
's IC constraint. Note that the IC constraint implies positive profits for firm 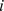 and assures its participation.

### Lemma 4

*The optimal stationary contract with reputation when quality is noncontractible,* 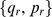 *, has the following features: (i) the IC constraint* 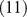 *is binding, hence firms get positive profits; (ii) the level of quality* 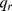 *belongs to interval* 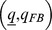 *; (iii) consumers accept the contract. In symbols:*

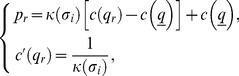
(18)where 
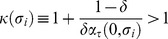
 and 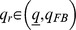.

### Proof

The problem to be solved is:
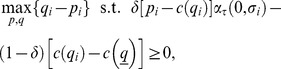
(19)where the constraint is 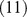 after rearrangement. The Lagrangian is



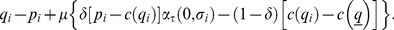
(20)The first order conditions with respect to 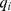 and 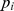 are:
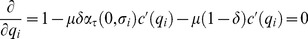
(21)


and
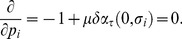
(22)


The constraint is binding at the optimum. Substituting 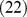 into (21) and rearranging yields
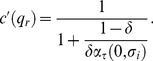
(23)


Note that
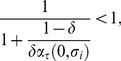
(24)hence 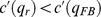, which implies 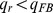. Finally, 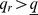 because 
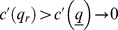
. Solving the binding constraint for 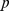 yields 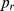. ▪

Recall that welfare 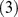 is maximum at 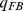 and, given its strict concavity due to 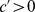 and 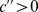, increasing in 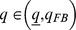. Since 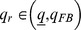, the welfare is larger under contract 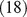 than contract 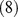: reputation mitigates the problem of unexploited gains from trade due to quality noncontractibility.

Finally, we investigate the relation between the quality level and the market share 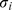 at the optimum described by 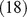.

### Lemma 5

*Quality level* 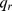 *increases with market share* 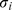 *.*

### Proof

The second equation of 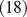 implies that
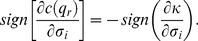
(25)


In turn 
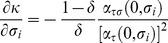
, which is negative under Assumption 3. As a result, 
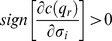
 and, given 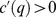, 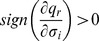. ▪

The result of Lemma 5 relies upon Assumption 3, according to which firms with greater market share are more easily discovered after cheating. As a consequence, they are also more credible when offering higher quality.

We now turn to the investigation of the strategic interaction among firms and consumers. We study the following infinitely repeated game with free entry:

- firms decide whether to enter the market;
- firms compete à la Bertrand on 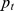 and 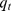 (recall that the level of quality 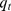 is promised by firms);
- consumers either select the preferred contract or do not purchase;
- firms select an actual level of quality for each consumer;
- Nature selects the public signal;
- the game starts again from stage (a).

We solve the game by focusing on symmetric Perfect Public Equilibria (PPEs, henceforth) in pure strategies. Symmetry means that all firms have the same market share. This implies that 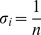.

Before proceeding we introduce the following

### Definition 1

*Quality level* 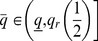 *is a minimum socially accepted quality standard, where* 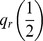 *denotes the quality level computed in Lemma 5 when just two symmetric firms are active in the market, that is,* 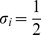 *,* 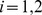 *.*

It seems reasonable to suppose the existence of a social convention on acceptable quality above the minimum 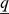. For instance, market shares of online insurance companies experienced very little growth in many economies since their appearance [20]. Given that online companies generally offer lower quality than traditional competitors, their poor performance may be due to the existence of a social convention on the quality of insurance policies, which prevents many potential customers from buying policies online.

We state the following

### Proposition 1

*There exists a PPE of the infinitely repeated game described above with the following features:*

1. *the equilibrium number of firms is*

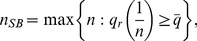
(26)
*where* 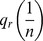 *is the equilibrium quality determined in* 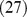 *;*
2. *on the equilibrium path all firms offer contract* 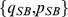 *characterized by:*

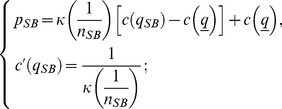
(27)
3. *off the equilibrium path, that is if* 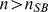 *, all firms offer contract* 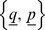 *of Lemma 2;*
4. *consumers accept contract* 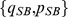 *if* 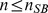 *, and accept contract* 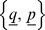 *if* 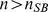 *; they refuse any other contract;*
5. *consumers refuse any contract from firm* 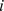 *after receiving the public signal, in which case firm* 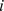 *exits the market.*

### Proof

(i) Point 5. According to the equilibrium strategy consumers do not buy upon receiving the public signal from firm 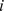. Each consumer expects then all the other clients not to buy from firm 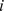 and anticipates that firm *i*'s market share will tend to zero. As a result, each consumer also anticipates that a poor quality level will be actually supplied by firm 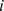. In symbols, if 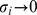, 
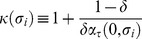
, computed in Lemma 4, becomes large because 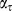 becomes small under Assumption 3. In that case, 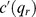 in 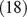 tends to zero, hence 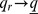 given that 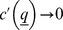. Consumers prefer thus to buy from another firm and firm 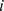 is forced to exit the market.

(ii) Point 4. First focus on the case 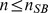. Contract 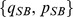 in 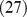 satisfies with the equality the IC constraint 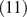, hence consumers accept it since they get the maximum utility. To prove it, note that two possible deviations are available to any firm 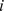: offering a contract with either (a) better or (b) worse conditions or the clients. Yet in case (a) the IC constraint is violated. In case (b) consumers simply refuse to buy.

Consider now the case 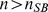. If all firms offer contract 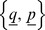 in 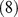, the clients accept it since it is the maximum they can get when quality is bounded to 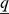. Again, two possible deviations are available to any firm 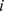. If a contract with better conditions for the clients is proposed by firm 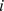, its participation constraint is violated. If a contract with worse conditions is proposed by firm 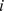, consumers simply refuse to buy.

(iii) Point 3. To prove that in each period 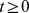 contract 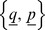 is an equilibrium contract when 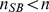, recall that firms make zero profits under this contract. The reasoning of Point 4 proves that any other contract would be refused by consumers, hence firms would make zero profits zero profits as well. We conclude that there is no strictly profitable deviation.

(iv) Point 2. To prove that in each period 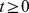 contract 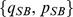 is an equilibrium contract for any 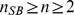, recall that such a contract satisfies the IC constraint 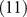 with equality. If all firms offer it, consumers accept and firms get 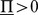 on each contract stipulated at each time 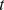. The reasoning of Point 4 proves that any other contract would be refused by consumers, hence there is no profitable deviation.

(v) Point 1. Suppose first 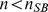 firms enter with 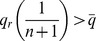. According to Lemma 5 at least an additional firm can enter and offer the following contract



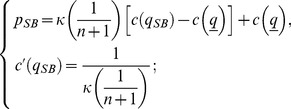
(28)Such an offer would be accepted given 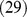, hence the entrant would end up with positive profits. We conclude that 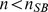 cannot be an equilibrium of the initial entry stage.

Now suppose 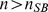 firms enter. This implies 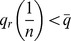 given Lemma 5, hence consumers predict that the market share of any firm offering a contract with 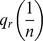 will be zero. This is because of the consumers' beliefs 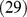, according to which no consumer would accept a contract with quality lower than the socially accepted quality standard 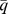. Following the reasoning of Point 5, any consumer knows then that only contracts promising minimum quality 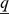 are incentive compatible for firms with zero market share. As a result, firms compete à la Bertrand by offering contracts with 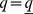, in which case they make zero profits as stated by Lemma 2. Therefore, entry when 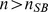 is not a strictly profitable strategy for outside firms.

We conclude that the equilibrium number of firms is 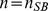. ▪

We remark that the equilibrium contract 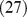 is driven by consumers' beliefs, an example of which is given by
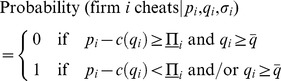
(29)


According to 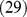, consumers anticipate that firm 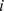 will not cheat when offering 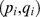 if the contract satisfies the IC constraint 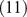 for any given 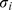 and if the promised level of quality is nonlower than the socially accepted quality standard 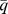. On the contrary, if 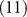 is not satisfied and/or firm 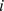 offers less than 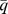, consumers believe that firm 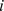 wants to cheat all of them. Such behavior on the part of consumers can be explained as follows.

Suppose at the equilibrium a firm decide to offer 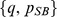 with 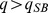. In this case its IC constraint is violated, hence consumers correctly anticipate that they will be cheated. Alternatively, suppose the firm offers 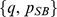 with 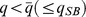. In this case the firm's IC constraint is fulfilled. Yet if any other competitor is fulfilling the socially accepted quality standard by offering 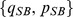, each individual consumer, in conformity with the equilibrium strategy, point 1 of Proposition 1, expects that none of the current clients will accept the contract proposed by firm 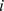. She thus anticipates that firm *i*'s market share will go to zero. In that case, Lemma 5 ensures that a poor quality level will be actually supplied by firm 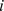. In symbols, if 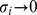, 
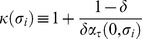
 in Lemma 4 becomes large because 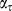 becomes small under Assumption 3. In that case, 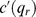 in 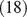 tends to zero, hence 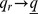 given that 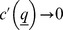. By anticipating this scenario, each consumer finds it rational to turn to any other competitor who offers 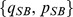. This reasoning clarifies why the social convention is fulfilled at equilibrium, with the effect that high quality is provided by the competitive firms.

## Discussion

We discuss the two most important results of Proposition 1. Lemma 5 ensures that the optimal quality 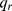 decreases with the number of active competitors 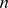. Therefore, firms enter the market until quality is non-lower than the acceptable standard 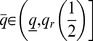. Put differently, the equilibrium number of firms is finite; at least two firms are active in the market, 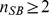, given that 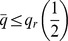. Note that if we let 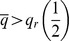, there would be equilibria with either zero or only one firm entering the market. However, in the latter case, the equilibrium contract would be different from 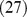, because the firm would act as a monopolist.

As a consequence, (i) the equilibrium quality 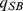 is higher than the minimum, 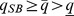, thanks to the social convention; (ii) the firms' IC constraint is binding at equilibrium, hence firms make positive profits on each contract,
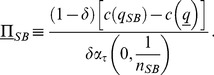
(30)


It is worth noting that the equilibrium described in Proposition 1 is not unique. Indeed, it hinges upon consumers' beliefs 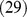. These beliefs may obviously be built in different ways, which would give rise to different equilibria. Even focusing on beliefs 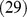, different equilibria are sustained depending on the value of 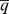.

In order to better understand our results, we consider explicit functional forms for the quality cost 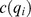 and the public signal probability 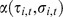. We then provide numerical simulations by assigning opportune values to the relevant parameters.

We let 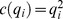 and 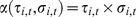: note these two functions fit with all the properties specified in the text. In addition, we let 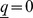, so that 
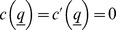
. One can check that the equilibrium contract when quality is contractible, computed in Lemma 1, becomes
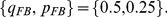
(31)


The equilibrium contract when quality is instead noncontractible, computed in Lemma 2, can be rewritten as
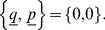
(32)


In turn, the IC constraint of Lemma 3 becomes
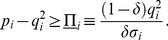
(33)


Finally, the optimal stationary contract with reputation when quality is noncontractible, computed in Lemma 4, can be rewritten as
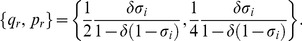
(34)


**Figure 1:**
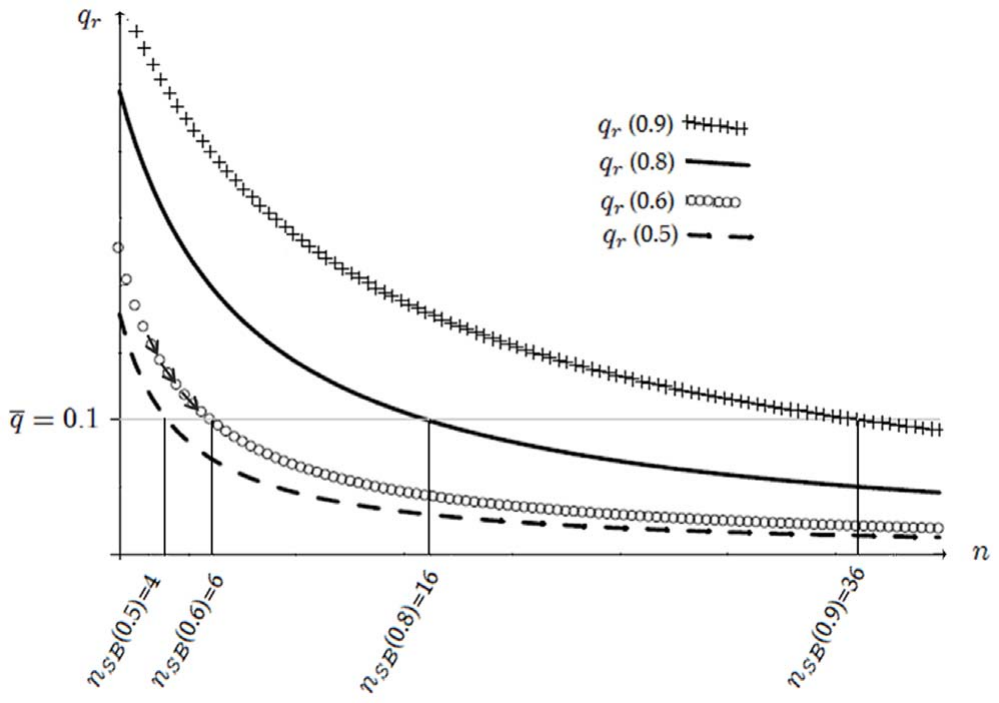
Constrained optimal quality 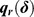 as a function of the number 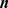 of active firms. Quality 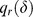 decreases for any 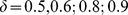 as new firms enter the market (as 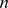 increases). Entry is blocked when quality reaches the social standard, 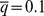 in the graph. Focus, *e.g.*, on 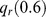, the constrained optimal quality when 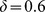: only six firms can enter the market, 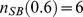, because a seventh competitor would supply lower quality than 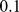.



Recalling that all firms have the same market share at our symmetric equilibrium, *i.e.*, 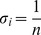, we present in Figure 1 the constrained optimal quality,

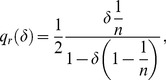
(35)as a function of the number 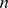 of active firms. We consider four different values of the discount factor 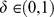, 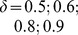. Note that interval 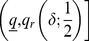, introduced in Definition 1 to establish the range of values that the socially accepted quality standard 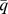 can take, becomes 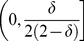, with 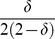 increasing in 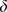. More precisely, the upper bound 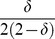 is equal to 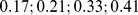 for 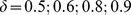, respectively. Accordingly, we let 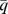 be equal to 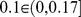.

Figure 1 confirms that 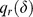 decreases with 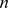, or, equivalently, increases with 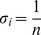, as stated in Lemma 5. The negative relation between 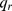 and 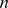 holds true for any 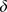. Following point 1 of Proposition 1 and recalling that 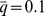, we can state that the equilibrium number of firms is 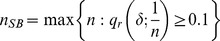. We get 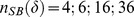 for 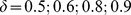, respectively. The intuition for this result is as follows. Focus, *e.g.*, on 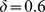. In that case, the equilibrium quality level would become strictly lower than the social standard 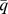 if at least 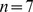 firms were active in the market. In symbols, 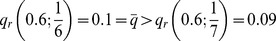. Note also that 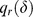 increases with 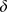 for any given 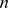. This is because a larger discount factor denotes a situation where the firms care increasingly about future profits. In this case, they are willing to offer higher quality because of the augmented cost of cheating clients. Consequently, more firms can enter the market as 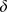 augments.

The above analysis confirms that at the equilibrium described by Proposition 1, (i) the quality level is higher than the minimum, 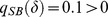; (ii) firms' per-contract profits are positive, 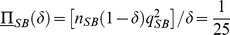, given that the IC constraint (33) is binding. Finally, note that the equilibrium quality is always below the efficient level. In symbols, 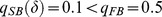. Therefore reputation increases quality from 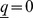 to 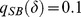, but it is not able to restore full efficiency since consumers must pay an informational rent to the producers.

**Figure 2:**
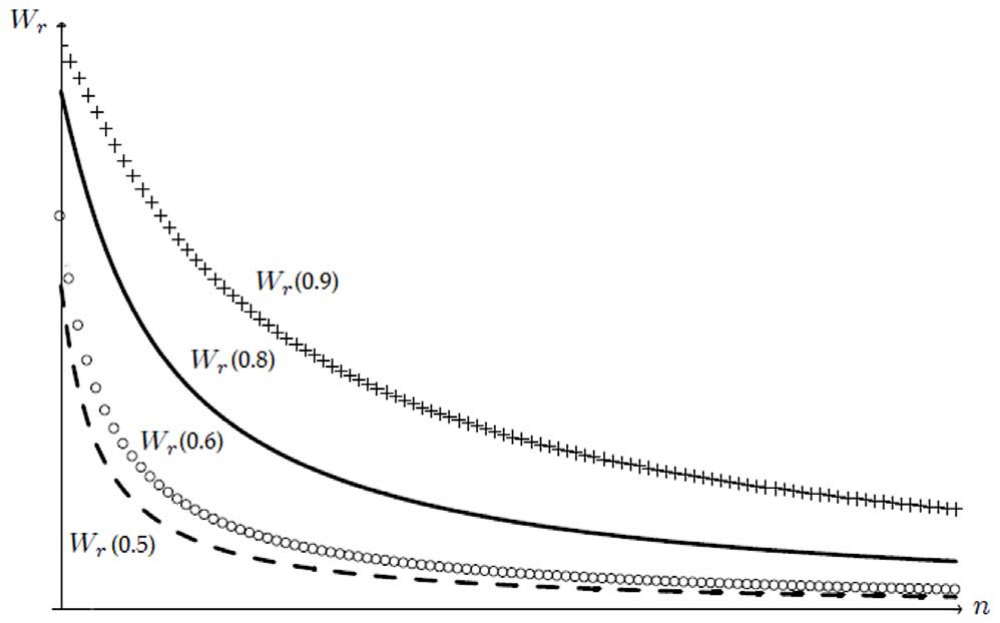
Constrained optimal welfare 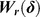 as a function of the number 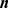 of active firms. Welfare 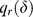 decreases for any 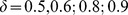 as new firms enter the market (as 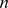 increases) because decreasing quality is offered.

To provide an additional interesting insight, we plug 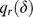, as in (35), into 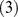 to get the value of welfare at the constrained optimum, 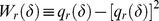. In Figure 2 we depict 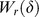 as a function of 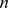 and of, for the sake of comparison, 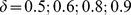. It is worth noting that 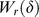 is always decreasing in 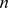. As a result, a larger value of the social standard 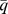 affects positively the welfare because it commands an increase in the equilibrium quality 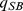 and, according to Lemma 5, a reduction in the equilibrium number of active firms.

To conclude our analysis, we are interested in checking the robustness of the equilibrium results concerning high quality and firms' positive per-contract profits. To this aim, we investigate the following three extensions/modifications of our framework.

### (a) Private Signal

We generalize our framework by introducing a private signal about quality of the good. More precisely, we suppose that a fraction 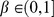 of the clients cheated by firm 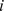 at time 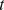, 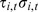, receive a private signal on top of the public one, in which case they do not buy anymore from firm 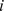. Two aspects of this formalization are worth remarking. (i) If no clients are cheated, 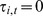, no private signal is conveyed because 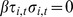. (ii) 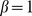 denotes a situation where all cheated clients get the signal, that is, they are able to perfectly observe the quality level after the contracts are implemented.

Our findings of Proposition 1 are robust to this richer specification because Lemma 6 below proves that the IC constraint 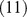 continues to hold true.

### Lemma 6

*When the private signal described above is received by the clients together with the public signal, firm* 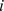 *decides not to cheat any consumers if and only if the IC constraint* 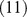 *holds true.*

### Proof

We prove that the result of Lemma 3 is robust to a single-period deviation, that is, firm 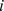 setting 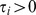 at time 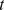 and 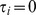 from 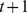 onward, when the private signal is taken into account. The discounted value of firm *i*'s profit at time 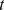, 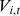 in (10), becomes
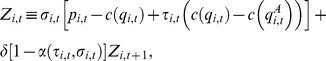
(36)with
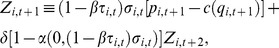
(37)after setting 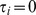 from 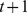 onward. Note that firm *i*'s market share at 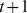, 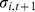, is equal to 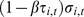 for a fraction 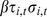 of customers leaves upon receiving the private signal.

One can check that 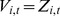 if 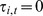 since no consumer receives the private signal. By contrast, if 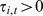, 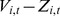 can be written as
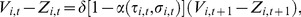
(38)where 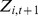 is given by 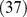 and



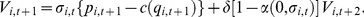
(39)Note that 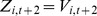 since 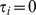 from 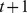 onward. Moreover,
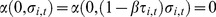
(40)according to Assumption 1. It follows that
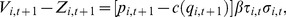
(41)which is positive. Hence 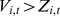 at 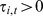. Lemma 3 proves that 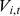 is maximized at 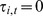. Since 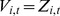 at 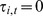 and 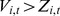 at 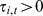, we can conclude that 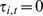 maximizes also 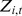. ▪

The intuition for this result is straightforward. For any given fraction of cheated consumers, the probability that firms lose clients is greater when consumers receive an additional signal about quality. By contrast, if firm 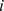 behaves, 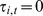, no private signal is conveyed, hence time-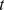 discounted value of firm *i*'s profit boils down to (10). As a result, any firm 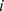 behaves if and only if the IC constraint 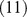 is fulfilled, in which case the equilibrium results are as in Proposition 1. Remark that a different equilibrium notion should be adopted if we solved the repeated competition game of Section "Results" with both public and private signal. Since the firms' quality level can now be imperfectly observed also through a private signal, Perfect Bayesian Equilibrium, and not PPE, is the proper solution concept.

### (b) Relaxing Assumption 1

Assumption 1 states that non-cheated consumers cannot send signals of bad quality. We relax it by considering an alternative public signal probability, 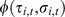, where a positive probability of sending a signal of bad quality exists even if firm 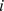 does not cheat any client at time 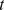, *i.e.*, 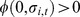. In that case, one can easily check that the IC constraint 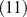 must be rewritten as
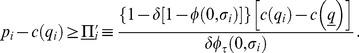
(42)


At the equilibrium of the repeated competition game, where the new IC constraint (42) is binding, a finite number of firms is active in the market, their profits on each contract are positive, and the quality level is above the minimum thanks to the social convention, as stated by Proposition 1.

To see this, we rely on the numerical simulations introduced above and let 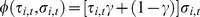, with 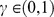. Note that 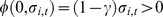. Moreover, 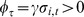, 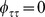, and 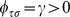 in conformity with Assumptions 2 and 3. By letting, *e.g.*, 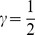, one can easily check that the IC constraint of Lemma 3 becomes
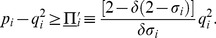
(43)


In that case, the optimal stationary contract computed in Lemma 4 can be rewritten as
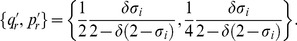
(44)


**Figure 3:**
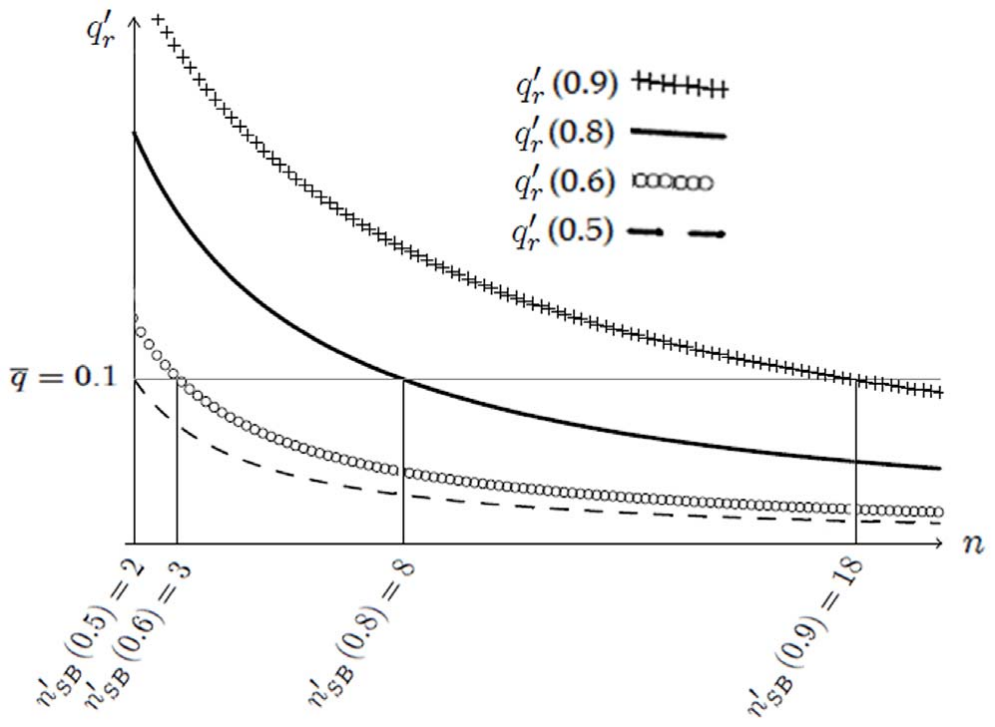
Constrained optimal quality 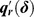 as a function of the number 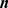 of active firms when false and/or erroneous signals of bad quality can be conveyed. Quality 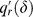 decreases for any 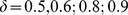 when new firms enter the market, as in Figure 1. Entry is blocked when quality reaches the social standard, 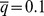 in the graph. Focus, *e.g.*, on 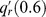, the constrained optimal quality when 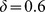: only three firms can enter the market, 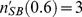, because a fourth competitor would supply lower quality than 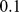.



In Figure 3 we present the constrained optimal quality,

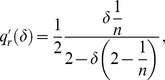
(45)as a function of the number 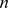 of active firms and of 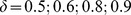. Note that interval 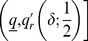 introduced in Definition 1 can be rewritten as 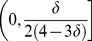, with 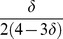 increasing in 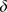 and equal to 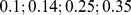 for 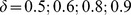, respectively. Accordingly, we let the socially accepted quality standard 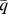 be still equal to 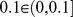.

Figure 3 confirms the result of Lemma 5: 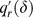 decreases with 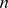. Recalling that 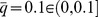, one can check that the equilibrium number of firms is 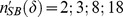 for 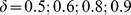. As a result, (i) the quality level is higher than the minimum, 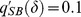 for any 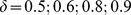, (ii) firms' per-contract profits are positive, 
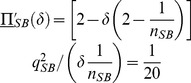
. One can also check that 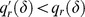 for any given 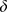 and 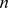: when non-cheated consumers may send signals of bad quality, the firms offer lower quality. This is because their profits are negatively affected by the increased probability that the signal is transmitted.

### (c) Relaxing Assumption 3

Assumption 3 states that bigger firms are more easily discovered when they cheat. We relax it by considering an alternative public signal probability, 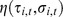, with 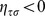: smaller firms are more easily discovered when they cheat. In that case, the result of Lemma 5 reverses in that quality level 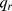 becomes decreasing in market share 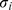. Put differently, quality is increased by entry of new firms. Entry is thus not blocked by the existence of a social convention with the effect that a huge number of firms is active in the market, *i.e.*, 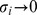 for any firm 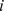. At the equilibrium of our repeated competition game, where the following new IC constraint is binding,
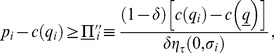
(46)the quality level is greater than the minimum, 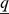, and firms get positive profits since both the numerator and the denominator of 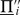 are positive when 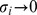.

**Figure 4:**
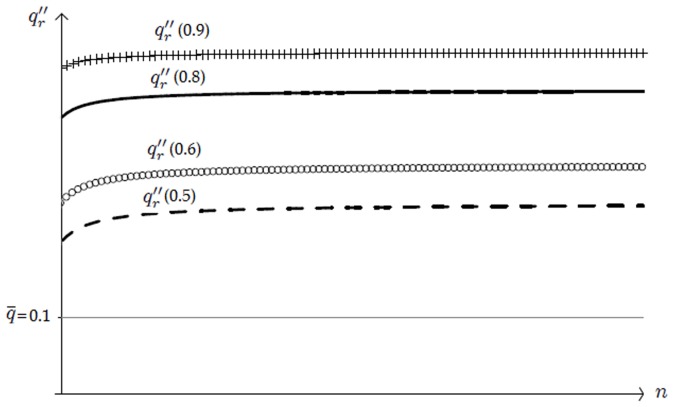
Constrained optimal quality 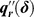 as a function of the number 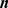 of active firms when smaller firms are more easily discovered upon cheating. Quality 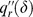 increases for any 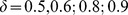 when new firms enter the market, unlike the scenarios described in Figures 1 and 3. As a result, the equilibrium level of quality is higher than the minimum.

Again we resort to the above numerical simulation to illustrate this result and we let 
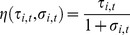
. Note that 
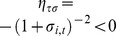
. Moreover, 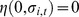, 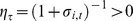, and 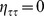 in conformity with Assumptions 1 and 2. One can easily check that the IC constraint of Lemma 3 becomes
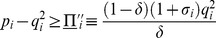
(47)and that the optimal contract of Lemma 4, can be rewritten as



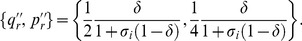
(48)In Figure 4 we present the constrained optimal quality
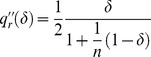



as a function of the number 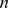 of active firms and of 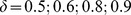. For the sake of comparison, we let the socially accepted quality standard 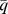 be still equal to 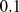.

Quality 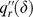 increases with 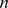: the result of Lemma 5 reverses, as stated above. Since entry is not blocked by the existence of a social standard on quality, the equilibrium number of firms is 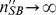. At the equilibrium described by Proposition 1, (i) the quality level is thus higher than the minimum, 
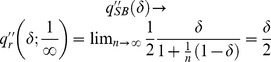
, (ii) firms' per-contract profits are positive, 
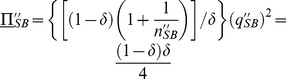
.

A major lesson comes from the three above extensions. The equilibrium results of high quality and firms' positive profits are robust to the introduction of a private signal about quality and to alternative specifications of the public signal probability.

## Conclusion

In this paper we tackled the issue of non-contractible quality provided by competitive symmetric firms. Consumers infer future levels of quality both from past levels and from current prices. We initially characterized the equilibrium contract in a static context and then showed that firms have no incentive to provide high quality. We then introduced reputation and demonstrated that firms end up with positive profits and supply high-quality goods. This provides a simple solution to the important objection raised by Joseph Stiglitz [7]. We finally proved that our results are robust to three different modifications of the framework.
